# A statistical assessment of population trends for data deficient Mexican amphibians

**DOI:** 10.7717/peerj.703

**Published:** 2014-12-16

**Authors:** Esther Quintero, Anne E. Thessen, Paulina Arias-Caballero, Bárbara Ayala-Orozco

**Affiliations:** 1 Subcoordinación de Especies Prioritarias, Dirección General de Análisis y Prioridades, Comisión Nacional para el Conocimiento y Uso de la Biodiversidad, Mexico D.F., Mexico; 2 The Data Detektiv, Waltham, MA, USA; 3 The Ronin Institute for Independent Scholarship, Montclair, NJ, USA

**Keywords:** Mexican amphibians, Statistical assessment, Encyclopedia of life, Random forests, Data harvest, IUCN categories

## Abstract

**Background.** Mexico has the world’s fifth largest population of amphibians and the second country with the highest quantity of threatened amphibian species. About 10% of Mexican amphibians lack enough data to be assigned to a risk category by the IUCN, so in this paper we want to test a statistical tool that, in the absence of specific demographic data, can assess a species’ risk of extinction, population trend, and to better understand which variables increase their vulnerability. Recent studies have demonstrated that the risk of species decline depends on extrinsic and intrinsic traits, thus including both of them for assessing extinction might render more accurate assessment of threats.

**Methods.** We harvested data from the Encyclopedia of Life (EOL) and the published literature for Mexican amphibians, and used these data to assess the population trend of some of the Mexican species that have been assigned to the Data Deficient category of the IUCN using Random Forests, a Machine Learning method that gives a prediction of complex processes and identifies the most important variables that account for the predictions.

**Results.** Our results show that most of the data deficient Mexican amphibians that we used have decreasing population trends. We found that Random Forests is a solid way to identify species with decreasing population trends when no demographic data is available. Moreover, we point to the most important variables that make species more vulnerable for extinction. This exercise is a very valuable first step in assigning conservation priorities for poorly known species.

## Introduction

Efforts to assign risk categories to species are one of the first steps in planning conservation strategies. One of the most recognized efforts to assign risk categories to species is that of the International Union for Conservation of Nature (IUCN), which recognizes seven different extinction risk categories for evaluating species: two of them are for species that are already extinct (Extinct and Extinct in the wild), three are for those considered as threatened (Critically Endangered, Endangered, and Vulnerable), two are for those species that are not yet threatened (Near Threatened and Least Concern), whereas the last one is for those species about which not enough information is known to perform an evaluation (Data Deficient). The IUCN also lists species that have not yet been evaluated (Not Evaluated).

IUCN’s criteria for assigning a threat category to a species are “quantitative in nature” meaning that punctuation is given for each category according to a given criteria, but both the quality and the uncertainty attached to any data in the evaluation vary. Estimates, inferences, projections and suspected facts based on related data are acceptable, as long as they can be supported and specified in the documentation. Moreover, time lag between assessments, human errors associated to them, and the lack of knowledge of the specialists on any given criteria may cause bias to the results of the assessments.

The Data Deficient category (DD) is assigned to those species in which the available data is not enough to determine a threat category, not even indirectly: for example, through the status of their habitat or other causal factors (IUCN, 2012). Only approximately 75,000 out of the 2 million described species are evaluated by the IUCN, and one sixth of them are Data Deficient (http://www.iucnredlist.org/about/summary-statistics), with 25% of all amphibians classified as such (Stuart et al., 2004). By lacking a threat status, Data Deficient species are not taken into account for conservation programs, potentially placing them at a higher risk of extinction. Thus, it is our objective to evaluate a statistical method to obtain the population trend of some species categorized as DD as a first step in prioritizing conservation actions. Our motivation is to test a more automated method of evaluating risk that can use a wider variety of available data and still give accurate results.

Recent studies have demonstrated that the risk of species decline depends on the specific threats they face, such as habitat loss, presence of invasive species, and pathogens (extrinsic traits), and the species’ own biological ability to cope with these threats (intrinsic traits), such as clutch and body size. Thus, including intrinsic traits along with extrinsic threats for assessing extinction might render a more accurate assessment of threat (Murray et al., 2011; Tingley, Hitchmough & Chapple, 2013), and thus improve allocation of resources (Cardillo & Meijaard, 2012).

Among all terrestrial vertebrates, amphibians are the group with more “rapidly declining species” recognized (Stuart et al., 2004). Mexico has the world’s fifth largest population of amphibians (Frost, 2014; Ochoa-Ochoa & Flores-Villela, 2006) with around 375 documented species, and the second country with the highest quantity of threatened amphibian species, 211 (IUCN, 2014) and whereas 38 (10%) amphibian species are currently listed as DD (IUCN, 2014) due to the lack of data (i.e., geographic distribution, threats, population status, etc.) to assign them to a risk status. The development of tools that allow assessing species’ risk of extinction in the absence of specific demographic data, as well as understanding which variables increase vulnerability to extinction could improve our ability to prioritize; focus, and or direct conservation actions in between field assessments or other types of research can be carried out.

The first Global Amphibian Assessment (Stuart et al., 2004) found that amphibian declines are not random, but are associated to ecological traits (i.e., stream associated species), geographic distribution (i.e., montane areas in the Neotropics, Australia and New Zealand), and specific taxonomic groups (i.e., Leptodactylidae, Bufonidae, Ambystomatidae, Hylidae, and Ranidae). Moreover, they divided the causes of decline in three groups: over-exploitation, defined as those declining due to heavy extraction (concentrated in species in East and Southeastern Asia); reduced habitat, defined as those that were suffering from extreme habitat loss (concentrated in Southeast Asia, West Africa, and the Caribbean); and enigmatic declines, those that are declining even though suitable area remains (restricted mostly to South America, Mesoamerica, Puerto Rico and Australia). Enigmatic declines were found to be positively associated with streams at high elevations in the tropics, and chytridiomycosis emerged as the most likely culprit.

Chytridiomycosis is a fungal disease caused by *Batrachochytrium dendrobatidis*, and has been related to the decline of at least 43 species of amphibians in Latin America (Lips et al., 2006). In México, there is an association between higher elevations (from 939 to 3,200 m) and the prevalence of the infection, but does not seem very common throughout tropical rain forests or lowland deserts (Frías-Alvarez et al., 2008). The reason for this marked preference for high areas with temperate climates may be that the optimal range of growth for this fungus is between 17 and 25 C (Piotrowski, Annis & Longcore, 2004; Longcore, Pessier & Nichols, 1999). Different surveys for the presence of chytridiomycosis in Mexico have found the presence of the fungus in different ecosystems, including those that have reported “enigmatic declines” in amphibian populations (Frías-Alvarez et al., 2008; Murrieta-Galindo et al., 2014). The finding by these authors suggests that chytridiomycosis, along with other threats, may be contributing to some of the amphibian declines.

In this study we harvested data for Mexican amphibians from the Encyclopedia of Life (EOL) and from published literature and used these data to assess the population trend of some of the Mexican species assigned to the DD category by the IUCN using Random Forests, a Machine Learning method algorithm that gives a prediction of complex processes and identifies the most important variables that account for the predictions (Breiman, 2001; Cutler et al., 2007; Murray et al., 2011). A recent assessment of DD mammals using and comparing multiple Machine Learning tools found that Random Forests perform very well for this type of predictions (Bland et al., in press). This is the first attempt to evaluate DD Mexican amphibians, and we hope to open the door to an improvement of the assessment of other groups for which we might lack demographic data. At the same time, we want to show how automatically harvesting data from online repositories can be used to answer biological relevant questions.

## Methods

### Selecting traits for the analysis

In order to assess the population traits of those species listed as Data Deficient, we selected previously identified intrinsic traits that can predispose species to a greater degree of vulnerability, as well as a series of extrinsic traits that have been associated to amphibian decline (Lips, Reeve & Witters, 2003; Stuart et al., 2004).

The extrinsic traits in our analysis were: habitat use; habitat loss/degradation, one of the biggest concerns for biodiversity (Assessment, 2005; Brooks et al., 2002; Frías-Alvarez et al., 2008; Groombridge, 1992; Lips, Reeve & Witters, 2003; Parra-Olea, García-París & Wake, 1999; Wyman, 1990); desiccation of bodies of water, which can take place independently of habitat loss and/or degradation (treated separately from other threats due to the high dependence of many amphibian species on them); presence of introduced species; presence of pollution; impact of known climatic fluctuations, as some authors have speculated that changes in precipitation patterns could indirectly contribute to the decline of some amphibian species, especially in those more sensitive to moisture changes (Carey et al., 2001; Kiesecker, Blaustein & Belden, 2001; Pounds & Crump, 1994); harvest for pet trade; presence of chytridiomycosis; and presence of other diseases that may decimate populations. The intrinsic traits selected for our analysis were snout-vent length, as species with larger body size have been observed to have a greater chance of decline than those with a smaller one (Lips, Reeve & Witters, 2003), and ova and clutch size as they are indirect measures of reproductive effort and reproductive rates, respectively, both linked to biological vulnerability, development type and breeding habitat, as species that depend on aquatic habitats seem to be more vulnerable than terrestrial ones (Lips, Reeve & Witters, 2003). The intrinsic traits were used here as a way to understand life history, ecological preferences, and vulnerability of the analyzed species (Lips, Reeve & Witters, 2003; Murray et al., 2011). Finally, we used the published population trend data from the IUCN Red List (IUCN, 2014), as our goal was to determine the population trend for the DD species. Unfortunately, the terms associated to population trend (decreasing, increasing, stable, unknown) in the Red List assessments are not quantitative, but rather associated with expert knowledge. In this respect, we might not have an accurate account of how steeply or rapidly a population is declining. Moreover, we are aware of the caveat of using these kinds of data, which might not be completely objective or may even harbor mistakes, as the primary data source for our analysis. However, they are the only means we have to try to statistically assess the population trend for DD species, and we decided to use them for the purpose of this first exercise.

### Automated data harvesting from encyclopedia of life

Starting with a list of scientific names of amphibians in Mexico (Table S1), relevant data and text were harvested from EOL using TraitBank (CS Parr, N Wilson, K Schulz, P Leary, J Hammock, J Rice, RJ Corrigan Jr, 2014, unpublished data) and the EOL API respectively (Parr et al., 2014). API stands for Application Programming Interface. An API is a piece of software that allows computers to share data without the need for repeated human action (http://en.wikipedia.org/wiki/Application_programming_interface). The code written for this project can be found at GitHub (https://github.com/diatomsRcool/MexicanAmphibians). Data from EOL TraitBank was retrieved by searching for taxon and measurement and downloaded as a .csv file. The EOL API was used to filter and harvest all relevant text based on relevant keywords (Table 1). The keywords were chosen based on commonly used phrases in a subset of EOL text. This process identified a subset of text for manual data extraction. Data from TraitBank and the text data objects were added to a master spreadsheet for analysis (Table S1). Data gathered for this study that was not already in TraitBank (see below) was placed in a Darwin Core Archive and uploaded into EOL TraitBank.

**Table 1:** EOL chapters and keywords used to filter and harvest relevant text data object for the study.

Trait type	EOL chapter	Keyword
Intrinsic	Size, reproduction, life cycle	Length, clutch, egg, breeding, development, reproduction, hibernation
Extrinsic	Distribution, habitat	Occur, range, inhabit, found, precipitation, wet, arid, dry, moist, temperature, temperate, tropic

### Other sources of literature

Data that were not available from EOL were obtained from the literature and cited in Table S2. Data for threats (habitat loss/degradation, introduced species, pollution, chytridiomycosis, climatic fluctuations, pet trade/harvest, desiccation of habitat, and other diseases), as well as for population trend (decreasing, increasing, stable, unknown) were obtained from the IUCN Red List (IUCN, 2014).

### Data preparation

A table was prepared with 302 rows and 16 columns (Table S1). Each row represented a species and each column represented a trait of that species. Examination of this master table revealed two traits (ova size and clutch size; Table 2) and four species (*Bolitoglossa chinanteca*, *Dermophis oaxacae*, *Eleutherodactylus marnockii*, and *Eleutherodactylus verruculatus*) to be particularly data deficient (defined as 10 or more missing traits). An additional species was identified as being introduced (*Eleutherodactylus planirostris*). These traits and species were removed from the data set.

**Table 2:** Number of missing data points for each variable. The 30 “missing” data points for the IUCN status actually refer to the number of Data Deficient and Not Evaluated species.

Trait	Missing data
Snout-vent length	11
Habitat use	1
Ova size	276
Development	5
Clutch size	252
Habitat loss/degradation	4
Introduced species	4
Pollution	4
Chytridiomycosis	4
Climatic fluctuations	4
Pet harvest	4
Desiccation of habitat	4
Other diseases	4
IUCN status	30
Population trend	53

All traits were coded into numeric categories (Table 3). Snout to Vent length classifications followed (García & Ceballos, 1994). In habitat use, we distinguished permanent water associated from stream associated. Threats were treated as present (1) or absent (0). Chytridiomycosis was recorded as present in cases where it was reported as suspected. Missing data were represented by a blank cell. From this table, we prepared a csv file for missing data imputation in R, a programming language popular in bioinformatics and statistics (Ihaka & Gentleman, 1996). The scientific name, IUCN status, and population trend were removed before imputation.

**Table 3:** Numeric categories codes for the traits used in the study.

Trait	Category	Definition
Snout-vent length	1	Up to 69 mm
	2	70–120 mm
	3	121–171 mm
	4	More than 172 mm
Habitat use	1	Ephemeral pond associated
	2	Permanent water associated
	3	Stream associated
	4	Terrestrial
Development	1	Direct development
	2	Larval development
	3	Paedomorphic
Habitat loss/degradation	0	Absent
	1	Present
Introduced species	0	Absent
	1	Present
Pollution	0	Absent
	1	Present
Chytridiomycosis	0	Absent
	1	Present
Climatic fluctuations	0	Absent
	1	Present
Pet trade/harvest	0	Absent
	1	Present
Desiccation of habitat	0	Absent
	1	Present
Other diseases	0	Absent
	1	Present
Population trend	0	Decreasing
	1	Stable

### Missing data imputation

We used the mice package (Van Buuren & Groothuis-Oudshoorn, 2011) in R to impute missing values (http://cran.r-project.org/web/packages/mice/mice.pdf). Imputation is a statistical method for replacing missing data with a probable value based on other available information (Little & Rubin, 2002). The mice package uses Gibbs sampling of the rest of the data set to generate imputations (Casella & George, 1992). It is used to avoid bias that may be introduced by deleting cases with missing values. This was necessary because the randomForest function did not tolerate missing values. All data were imported into R as factors, i.e., categories. The Snout-Vent Length, Habitat Use, and Development Type were imputed as polytomous logistic regression (polyreg). The other traits were imputed using logistic regression (logreg). These settings are important for proper imputation. Missing Population Trend data were not imputed. Ten independent imputations were performed for each missing value. The final value was the mode of the 10 imputations. The data set before imputation can be found in Table S1. A summary of missing data can be found in Table 2 and Table S1. The data set after imputation can be found in Table S3. The data set that includes the imputed data was used for predicting the population trend for those species that were Data Deficient according to the IUCN evaluation.

### Predicting population trends

We used the randomForest package in R (Liaw & Wiener, 2002) to make predictions about the population trends for each species of amphibian (http://cran.r-project.org/web/packages/randomForest/randomForest.pdf). Random Forests is a machine learning method for classifying objects into categories by building a decision tree (Breiman, 2001). In this case, we wanted to classify species into stable *vs*. declining population trend. Random Forests generates multiple decision trees using a random selection of traits at each split in the tree. For x traits, the square root of x traits are used at each split. The final tree depends on the mode of the output of the individual decision trees. It is important to notice that the resulting population trend, just as in the case of that reported by IUCN, was not quantitative, but rather just a general tendency. We are aware that this lack of quantitative data may categorize two species with different natural histories and demographic trends in the same category, but we are also certain that this was the only way to proceed with the amount and nature of data that we have. At the same time, this general trend would result in exactly the very first step to assign conservation priorities to those species that at the moment are categorized as DD.

Preparing the randomForest was a three-step process: the first step was using training data, i.e., traits for those species with known population trends, to generate a random forest object. The second step was testing the random forest object on a separate data set of species with known population trends. The third step was using the random forest object to make predictions about population trends for those species listed as Data Deficient and Not Evaluated by the IUCN (IUCN, 2014).

The data set of species with known population trends was randomly divided in two to make a training set and a test set. The training data (including the imputed data) was read into R and given to the randomForest (Liaw & Wiener, 2002) function, which provided a random forest object as a result. To test the efficacy of the random forest for prediction, we removed the Population Trend data from the test set and made a prediction of population trend for comparison to the observed population trend (Table 4).

**Table 4:** Confusion matrix obtained using the randomForest and predict functions in the randomForest package (Liaw & Wiener, 2002) on the test data to predict population trend.

	PREDICTED	
**OBSERVED**	Decreasing	Stable
Decreasing	79	9
Stable	8	30

We added additional species, as needed, to the data set including all of the species with an unknown population trend (unknown data set) to balance the presence of categories for each trait, a requirement for making predictions. The unknown data set was read into R and given to the randomForest and predict functions in the randomForest package (Liaw & Wiener, 2002). The predict function in the randomForest package uses the Random Forest that we generated and tested to make predictions about the species with unknown population trend. Because the random forest is a decision tree, the predict function uses the traits in our data set to follow the decision tree to the final classification of population trend. There are multiple methods for selecting the traits that are important for building the decision tree. To ensure unbiased trait selection (Strobl et al., 2007), we used an additional method, the cforest (Hothorn et al., 2006; Strobl et al., 2008; Strobl et al., 2007) and predict functions in the party package (http://cran.r-project.org/web/packages/party/party.pdf), and compared trait selection to the randomForest function. Both functions use Random Forests to classify objects, but use different methods for aggregating the trees into one final decision tree (e.g., Hothorn et al., 2004). This is particularly important for this study because in cases where traits have a different number of categories, randomForest may be biased towards traits with more categories. To calculate *p* values for the branches in the tree, we used the ctree (Hothorn, Homik & Zeileis, 2006) function in the party package. This function tests for independence between the traits and the population trend and, where a relationship is found, implement a binary split and calculate a *p* value.

## Results and Discussion

**Figure 1:**
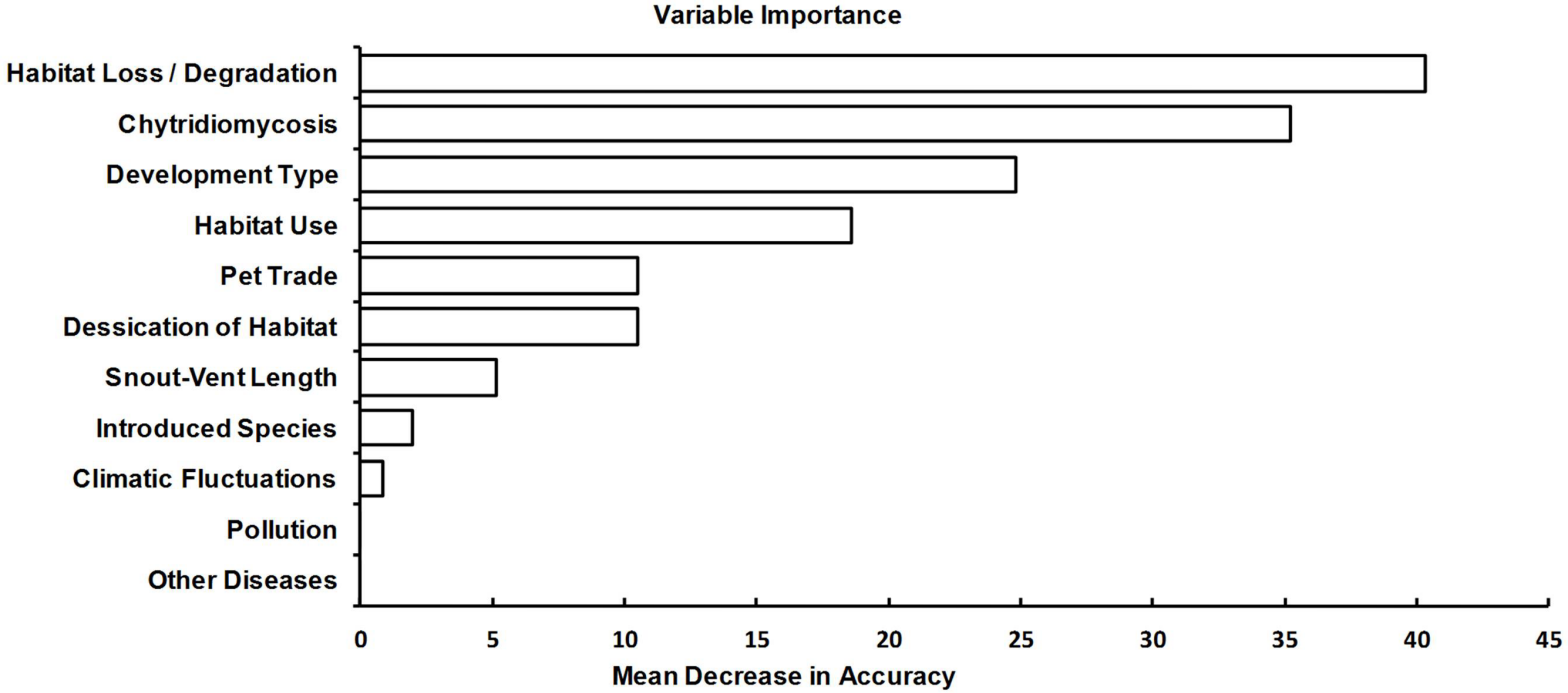
Relative importance of variables for predicting population trend. Bar graph showing the relative importance of all variables for predicting population trend. The individual variables are listed on the vertical axis. The horizontal axis shows the decrease in accuracy of the final result if the variable is removed. The removal of more important variables will result in a higher mean decrease in accuracy. For example, if Habitat Loss were removed from the analysis, the accuracy of the final result would drop by approximately 40%; therefore, it is an important variable.

Out of the 24 species classified as “Data Deficient” by the IUCN included in our analysis, 18 were predicted to be decreasing, and five were classified as stable (Table 5). In predicting Population Trend, the most important variables were Habitat Loss/Degradation, Presence of Chytridiomycosis, Development Type, and Habitat Use (Fig. 1). Random Forests and cforest (results not shown) show the same variables in the top four most important, which means that random Forests does not have variable selection bias in this analysis and these results are considered trustworthy from a statistical point of view. However, according to cforest, the importance of habitat loss dwarfs the importance of the other three variables. Independence between the trait and the population trend was tested by the ctree function using a Bonferroni test at each split in the tree. Habitat loss was the most significant (*p* < 0.001), followed by Development Type (*p* = 0.269), and the presence of Chytridiomycosis (*p* = 0.517). The fact that habitat loss/degradation was the most critical variable according to randomForest, cforest, and ctree (Fig. 1) concurs with the vast amount of information on the cause of species declines (Stuart et al., 2004; Assessment, 2005; Brooks et al., 2002; Frías-Alvarez et al., 2008; Groombridge, 1992; Lips, Reeve & Witters, 2003; Parra-Olea, García-París & Wake, 1999; Wyman, 1990).

**Table 5:** Predicted population trend for the 24 species classified as Data Deficient by the IUCN.

Species	Population trend predicted	Mexican red list
*Bolitoglossa oaxacensis*	Decreasing	–
*Bolitoglossa stuarti*	Stable	A
*Bolitoglossa zapoteca*	Decreasing	–
*Chiropterotriton mosaueri*	Decreasing	Pr
*Craugastor amniscola*	Decreasing	–
*Craugastor occidentalis*	Stable	–
*Craugastor pelorus*	Decreasing	–
*Craugastor taylori*	Stable	Pr
*Eleutherodactylus maurus*	Decreasing	Pr
*Eleutherodactylus pallidus*	Decreasing	Pr
*Eleutherodactylus teretistes*	Decreasing	Pr
*Exerodonta abdivita*	Decreasing	–
*Exerodonta bivocata*	Stable	–
*Lithobates lemosespinali*	Decreasing	–
*Pseudoeurycea amuzga*	Decreasing	–
*Pseudoeurycea maxima*	Decreasing	–
*Pseudoeurycea mixcoatl*	Decreasing	–
*Pseudoeurycea obesa*	Decreasing	–
*Pseudoeurycea quetzalanensis*	Decreasing	–
*Pseudoeurycea tlilicxitl*	Stable	–
*Ptychohyla acrochorda*	Decreasing	–
*Ptychohyla zophodes*	Decreasing	–
*Thorius insperatus*	Decreasing	–

**Notes.**

The categories on the 2010 official Mexican National Red List (NOM-Semarnat-059-2010) are as follows: E Extinct P Endangered A Threatened Pr Under special protection

Our randomForest analysis accurately identifies species with decreasing population trend (Table 4). In the test data there were 79 true positives, 9 false positives, 30 true negatives, and 8 false negatives (Precision = 0.897. Recall = 0.908; F1 score of 0.903 where 1 is a perfect score) for the “decreasing” category, which means that the method is likely to correctly flag a species as decreasing or stable. The errors are equally likely to be a false positive or negative.

Table 5 also shows the risk status according to the 2010 official Mexican National Red List for the Data Deficient species included in the analysis. Of them, only one, *Bolitoglossa stuarti,* is categorized as Endangered (A), while five of them (*Chiropterotriton mosaueri, Craugastor taylori, Eleutherodactylus maurus, Eleutherodactylus pallidus,* and *Eleutherodactylus teretistes*) are considered “Under Special Protection (PR)”, which is the lowest risk status of the List. Most amphibian species in the National Red List are currently placed on the lowest risk status due to a lack of Risk Assessment Method (MER according to the Spanish acronym) in order to comply with the precautionary principle. The other 17 species in our analysis have not been assessed at all at the national level and therefore are not listed. Of all the species on the Mexican Red List, only *Bolitoglossa stuarti*, and *Craugastor taylori* are predicted to have a stable population trend, so it would be advisable to assess the other 17 species for the next version of the official Mexican National Red List, as the National Red List is the only national policy instrument which foresees law enforcement in order to protect Mexican threatened species.

In a similar study of Australian amphibians, Murray et al. (2011) found that Habitat Use (ecological group) was the most important variable to determine population trend, followed by the presence of chytridiomycosis and *Gambusia*, a predatory fish (defined by spatial models of suitability). Contrary to what we did, these authors included range size (EOO), abundance, and testes mass, and the presence of *Gambusia*. Although our study and that of Murray et al. (2011) found different variables as the most important to determine population trend, both studies concurs with that of Murray et al. (2011) in that by using statistical methods one can not only get an account of the population trend of a given amphibian species, but also of the risk factors that are most pressing for the different ecological groups. In the case of Mexico however, habitat destruction is, by much, the most pressing risk factor, in as much as all the others are overshadowed by it.

## Conclusions

The use of Random Forests seems to be a solid way to identify species with decreasing population trends in the absence of demographic data in order to prioritize those in need of further assessment. The kind of exercise that we show here can be a first approach when planning conservation priorities, as some of the most endangered species might also be those for which most information is lacking. Moreover, this method has the advantage of not having to depend on aggregated museum locality data that may not have been properly curated by experts, as is the case for some assessment efforts (Hjarding, Tolley & Burgess, 2014).

Intrinsic factors, such as the ova and clutch size, can give important information about how life history can affect the population trend of a species. Unfortunately, the amount of data we had for those traits was so limited that we felt that including data that had mostly been statistically generated could introduce an extra bias to our analysis. The fact that so little information on the natural history of these endangered species is available is a major challenge that needs to be addressed to successfully prevent their extinction. In addition, our aggregated data set can be used to set data collection priorities to fill in gaps. Fortunately, as we show here, this lack of information should not deter the efforts to assess risk status and assign priorities to their conservation.

We would also like to call attention into the fact that the most recent global amphibian assessment was just conducted in September 2014. In this latest assessment the experts have expressed that, in fact, Chytridiomycosis is not the big concern it was thought to be 10 years ago, when the last global assessment was performed. This view, along a deeper knowledge of the complexity of the disease, is also mirrored in recent studies, which show that many amphibians have either developed resistance to the disease or some communities seem to be thriving even when the fungus is still present (Ellison et al., 2014; Gervasi et al., 2014; Langhammer et al., 2014; Louca, Lampo & Doebeli, 2014; McMahon et al., 2014; Scheele et al., 2014). However, experts also agree that habitat loss is still the biggest concern for amphibians and biodiversity in general (P Arias-Caballero, pers. comm., 2014).

Although it is not our claim that efforts like the one we present here should substitute for field research or other forms of assessments, we want to emphasize the importance of these exercises as part of the comprehensive effort to pinpoint research and assessment necessities to inform public policy and advance the conservation of the species. For instance, it would be very interesting to replicate this exercise with other DD species in different taxonomic groups. Moreover, we want to emphasize how important it is to use data archived in digital libraries such as EOL and BHL and statistical tools to answer questions that are difficult to tackle otherwise.

## Supplemental Information

10.7717/peerj.703/supp-1 Table S1 Mexican amphibian species and intrinsic and extrinsic traits used to assess the risk status for each species

10.7717/peerj.703/supp-2 Table S2 Data obtained from the literature

10.7717/peerj.703/supp-3 Table S3 Inputed values for each species trait

## Additional Information and Declarations

### Data Deposition

The following information was supplied regarding the deposition of related data: GitHub (https://github.com/diatomsRcool/MexicanAmphibians).
